# Purification, Identification and Molecular Docking of Immunomodulatory Peptides from the Heads of *Litopenaeus vannamei*

**DOI:** 10.3390/foods11203309

**Published:** 2022-10-21

**Authors:** Weiwei Jiang, Keyu Ren, Zhiyan Yang, Zhou Fang, Yan Li, Xi Xiang, Yishan Song

**Affiliations:** 1 College of Food Science & Technology, Shanghai Ocean University, Shanghai 201306, China; 2 National R&D Branch Center for Freshwater Aquatic Products Processing Technology (Shanghai), Shanghai 201306, China

**Keywords:** *L. vannamei* heads, immunomodulatory peptides, purification, identification, molecular docking

## Abstract

In order to realize the high-value utilization of *Litopenaeus vannamei* (*L. vannamei*) heads, immunomodulatory peptides were prepared from the enzymatic hydrolysate of *L. vannamei* heads, and the action mechanism of immunomodulatory peptides was determined by molecular docking. The results showed that six proteases were used to hydrolyze *L. vannamei* head proteins, with the animal protease hydrolysate exhibiting the highest macrophage relative proliferation rate (MRPR). The enzymatic products were then sequentially purified by ultrafiltration, Sephadex G-15 gel chromatography, identified by liquid chromatography-mass spectrometry (LC-MS/MS), and finally selected for six immunomodulatory peptides (PSPFPYFT, SAGFPEGF, GPQGPPGH, QGF, PGMR, and WQR). These peptides maintained good immune activity under heat treatment, pH treatment, and in vitro gastrointestinal digestion. Molecular docking analysis indicated that these peptides showed great binding to both toll-like receptor 2 and 4 (TLR2 and TLR4/MD-2), leading to immunomodulation. The discarded *L. vannamei* heads in this article are considered to be promising food-borne immunomodulators that contribute to enhancing the immune function of the body.

## 1. Introduction

The immune system covers immune defense, surveillance, and immune regulation and is linked to the etiology of various diseases. Currently, there are many drugs available to regulate the immune function in humans, including levamisole, cyclophosphamide, and macrolides [1], but most synthetic immunomodulatory drugs have toxic side effects on the body [2]. Therefore, synthetic immunomodulatory drugs are not conducive to prevention or long-term use, either from an economic or social perspective. In this regard, immunomodulators derived from natural foods have potential development value and significance owing to their effectiveness and safety for human health.

In recent years, research on bioactive compounds in marine organisms has become a popular topic. More recent attention has focused on the provision of bioactive peptides with different physiological functions, such as the dipeptidyl peptidase-IV (DPP-IV) inhibitory peptide [3], angiotensin converting enzyme (ACE) inhibitory peptide [4], and immunomodulatory peptide [5]. Previous research showed that immunomodulatory peptides could stimulate macrophage proliferation, enhance the phagocytosis of macrophages, and promote the secretion of cytokines and nitric oxide (NO) [6]. From *Alaska pollock*, Hou et al. extracted, purified, and identified Pro-Thr-Gly-Ala-Asp-Tyr, a peptide that markedly enhanced humoral, cellular, and non-specific immunity in immunosuppressed mice [7]. Mallet et al. enzymatically digested shark protein and purified it to produce small peptides that could enhance intestinal barrier function through cytokine production [8]. However, the extraction of immunomodulatory peptides from *Litopenaeus vannamei* has not been reported, particularly utilizing its discarded head.

*Litopenaeus vannamei* (*L. vannamei*), which belongs to the shrimp family and prawn animal genus, is one of the major species in the global shrimp-farming industry. China is a major shrimp-farming country [9], and during processing, 40–50% byproducts (heads, shells, and tails) are produced, which contain high-quality protein, chitin, minerals, etc., but are rarely utilized except for a few used as feed [10]. Moreover, a black spot can form and spread from the shrimp’s head. Although shrimp blackening is not detrimental to health, it affects consumer preference and the value of the product [11], so a large amount of this byproduct is wasted, resulting in the loss of bioactive components. Therefore, unutilized *L. vannamei* heads containing a high protein content [12,13] may be a potential source of immunomodulatory peptides.

In this paper, immunomodulatory enzymatic hydrolysates were prepared from discarded *L. vannamei* heads using different enzymes. The immunomodulatory peptides were purified using ultrafiltration and gel separation, and were identified by mass spectrometry. In addition, molecular docking was applied to elucidate the action mechanism of the immunomodulatory peptides. The aim was to provide a theoretical basis for the development of functional foods with immunomodulatory peptides from *L. vannamei* byproducts.

## 2. Materials and Methods

### 2.1. Materials

The *L. vannamei* was purchased from Shanghai Luchao Port. The clipped shrimp heads were stored at −80 °C until used for enzymatic hydrolysis. The RAW 264.7 cells were provided by Stem Cell Bank, Chinese Academy of Sciences. Compound protease (5.53 × 10^4^ U/g), neutral protease (1.33 × 10^5^ U/g), alkaline protease (7.51 × 10^4^ U/g), bromelain (1.04 × 10^5^ U/g), papain (8.29 × 10^4^ U/g), and animal protease (7.58 × 10^4^ U/g) were purchased from Tianjin Nuo’ao Technology Development Co., Ltd. (Tianjin, China); Lipopolysaccharide (LPS) and thiazolyl blue tetrazolium bromide (MTT) were purchased from Soleibo Biotechnology Co., Ltd. (Beijing, China); fetal bovine serum (FBS), penicillin/streptomycin double antibody, Dulbecco’s modified Eagle’s medium (DMEM), dimethyl sulfoxide (DMSO), phosphate-buffered saline (PBS), neutral red, NO, tumor necrosis factor-α (TNF-α), interleukin-6 (IL-6), interleukin-2 (IL-2), and interleukin-1β (IL-1β) kits were purchased from Shanghai Doushen Biotechnology Co., Ltd. (Shanghai, China). All other chemicals in this study were of analytical grade.

### 2.2. Preparation of L. vannamei Enzymatic Hydrolysate

To select the appropriate protease, frozen minced shrimp heads of *L. vannamei* were thawed at room temperature and then hydrolyzed using animal protease (50 °C pH 7.5), papain (50 °C pH 7.5), bromelain (50 °C pH 7.5), neutral protease (50 °C pH 7), alkaline protease (50 °C pH 9), and complex protease (55 °C pH 7.5) at a ratio of 3000 U/g. Deionized water was added to obtain a ratio of minced shrimp head/water of 1:5 (*w*/*v*) and the mixture was adjusted to the reaction pH with 0.1 M NaOH and HCl. All enzymes were hydrolyzed for 4 h. Then, the enzymatic hydrolysates were inactivated at 100 °C for 10 min and centrifuged at 4 °C at 10,000 r/min for 20 min. The supernatant was collected and freeze-dried to produce a lyophilized powder. The optimized conditions after using animal protease were chosen to be 1:6.1 (*w*/*v*) and 3500 U/g for the solid-liquid ratio and enzyme amount, respectively, and the enzymatic time was set to 5 h. The macrophage relative proliferation rate of the enzymatic hydrolysate was measured as a selection index, while the hydrolysis degree and molecular weight distribution of the enzymatic hydrolysate were determined.

### 2.3. Determination of Hydrolysis Degree (DH)

The DH of the enzymatic hydrolysates was analyzed as previously reported by Nielsen et al. [14].

### 2.4. Determination of Molecular Weight (MW)

The MWs of the enzymatic hydrolysates were assayed using a high-performance liquid chromatograph (Waters 2695-2489 instrument) equipped with a TSK gel 2000 SWXL column (300 × 7.8 mm, Tosoh, Tokyo, Japan) according to a previously reported method [15]. The MW standard curve was plotted based on the average retention times of the following five standards: Gly-Gly-Gly (189 Da), Gly-Gly-Tyr-Arg (451 Da), bacitracin (1450 Da), aprotinin (6511 Da), and cytochrome C (12384 Da). The mobile phase solvent was composed of acetonitrile, ultrapure water, and trifluoroacetic acid (45:55:0.1) at a flow rate of 0.5 mL/min and detected at 220 nm.

### 2.5. Ultrafiltration

According to the MW of the enzymatic hydrolysate, it was divided into four fractions: M1 (MW > 10 kDa), M2 (3 kDa < MW < 10 kDa), M3 (1 kDa < MW < 3 kDa), and M4 (MW < 1 kDa) through 10 kDa, 3 kDa, and 1 kDa ultrafiltration membranes (Millipore Corporation, Bedford, MA, USA), respectively. The four fractions were collected and lyophilized for use to compare their effects on MRPR.

### 2.6. Sephadex G-15 Separation

After G-15 gel pretreatment, the column was loaded and equilibrated, and the lyophilized powder obtained by ultrafiltration with the highest MRPR was dissolved in deionized water (30 mg/mL). Subsequently, 4 mL of the sample solution was injected into a Sephadex G-15 gel column (1.6 cm × 50 cm), eluted with deionized water at a rate of 0.5 mL/min, and the detection wavelength was 254 nm to pass the protein purification system (HDB-7, Huxi Analytical Instrument Factory Co., Ltd., Shanghai, China) to measure the absorbance of the sample elution curve, and finally, the automatic collector was set to 6 min/tube to complete the sample collection. The fractions were collected and then lyophilized to compare their effects on MRPR. The highest MRPR fraction was further evaluated for immunological activity.

### 2.7. Identification by Liquid Chromatography–Mass Spectrometry (LC-MS/MS)

Immunomodulatory peptide sequences were identified using LC-MS/MS according to the procedure of Xiang et al. with minor modifications [13]. After equilibrating the system with 95% solution A (0.1% formic acid in water), the sample solution was uploaded onto Zorbax 300 SB-C18 peptide traps, separated on a liquid chromatography column (0.15 mm×150 mm, RP-C18), and eluted by a linear gradient of 1% to 100% solvent B (acetonitrile solution of 0.1% formic acid) for 60 min. The MS/MS data were further processed using MaxQuant (version 1.5.5.1, Max Planck Institute of Biochemistry, Martinsried, Germany) by searching the corresponding UniProt_Penaeus_vannamei_26403_20220302 database. The resulting peptide sequences were scored by PeptideRanker (http://distilldeep.ucd.ie/PeptideRanker/, accessed on 20 June 2022) to predict whether each peptide possessed good biological activity.

### 2.8. Determination of the Relative Proliferation Rate of Macrophages RAW 264.7 (MRPR)

The macrophages were cultured in a medium (containing 90% DMEM medium, 10% FBS, and 1% double antibody) at 37 °C and 5% CO_2_. The MRPR was determined following to the MTT assay used by Xu et al. [16]. The density of the log-phase cells was adjusted to 1 × 10^5^ cells/mL, and 100 μL/well of cell suspension was seeded in 96-well plates and incubated overnight in an incubator (BB-150-CO2 incubator, Thermo Scientific, Waltham, MA, USA). The perimeter of the 96-well plate was filled with PBS. The old culture medium was then discarded, 200 μL of the sample solution was added (DMEM medium was used to dissolve the sample to the desired concentration), and control and blank groups were set up with DMEM medium. After incubating for 36 h, 200 μL of medium (containing 5% MTT) was added to each well instead of the original medium and cultured in the dark for 4 h. After the supernatant was discarded, 150 μL of DMSO was added, shaken for 20 min at room temperature, and the absorbance was detected at 490 nm by a microplate reader (ELx800, BioTek Instruments, Winooski, VT, USA). The MRPR (%) was calculated using the following formula:

Macrophage relative proliferation rate (%) = (A1 − A3)/(A2 − A3) × 100 (1)

where A1 and A2 represented the sample solution and DMEM medium with cells (sample and control group), respectively, and A3 represented DMEM medium with no cells (blank group).

### 2.9. Determination of Immune Activity Evaluation Index

#### 2.9.1. Determination of Phagocytosis

RAW 264.7 cells (1 × 10^5^ cells/mL, 100 μL) were inoculated in 96-well plates overnight. After treating the cells with samples for 24 h, 20 μL of neutral red staining solution was added to the medium, incubated for 2 h and discarded, and then washed twice with PBS. Next, 200 μL of red detection lysate was added and the cells were lysed on a shaker at room temperature for 10 min and measured at 540 nm.

#### 2.9.2. Determination of Released NO and Various Cytokines

RAW 264.7 cells (2 × 10^5^ cells/mL, 100 μL) were inoculated in 96-well plates overnight. The cells were then incubated for 24 h, with sample and cell supernatants collected separately. NO release was measured at 540 nm using the Griess reagent assay. The release of TNF-α, IL-2, IL-6, and IL-1β was measured using the kits. Then, 50 μL of sample-treated cell supernatant or indicator standard and 50 μL of biotin antigen were first added and reacted at 37 °C for 30 min. Then, after washing with washing solution, 50 μL of affinity-HRP was added for 30 min at 37 °C and washed again. After incubation with a color developer for 10 min, 50 μL of stop solution was added to determine the release of each cytokine at 450 nm.

### 2.10. Peptide Synthesis

The six immunomodulatory peptides identified by mass spectrometry were synthesized by solid-phase synthesis (commissioned from Dangang Biotechnology Co., Ltd., Wuhan, China), and the purity of the synthesized peptides was higher than 95%.

### 2.11. Stability Analysis of Immunomodulatory Peptides

For gastrointestinal digestion stability, computer simulated gastrointestinal digestion was first performed using the PeptideCutter (https://web.expasy.org/peptide_cutter/) (accessed on 20 June 2022) with a selection of pepsin (pH > 2) and trypsin. Then, six peptides (10 mg) were dissolved in 10 mL of distilled water, the pH was adjusted to pH 2.0 with 1 M HCl, and then 2% (*w*/*w*) pepsin was added and incubated at 37 °C for 2 h to simulate gastric digestion. The pH of the gastric digest was then adjusted to 5.3 with 0.9 M NaHCO_3_ solution and to pH 7.5 with 1 M NaOH solution. Following this, 2% (*w*/*w*) trypsin was added and incubated at 37 °C for 2 h to simulate intestinal digestion. After digestion, the digest was inactivated in a water bath at 100 °C for 10 min, centrifuged at 10,000 r/min for 10 min, and the supernatant was freeze-dried to obtain the gastric intestinal digest of each of the six synthetic peptides. The MRPR was determined using the sample without any treatment as the control.

To evaluate the thermal stability of the synthetic peptides, six peptide solutions (1 mg/mL) were heated at 40 °C, 60 °C, 80 °C, 100 °C, and 121 °C for 30 min, and then cooled and lyophilized to assess their immune activity. The unheated treated samples (room temperature: 25 °C) were used as controls to compare their MRPR with the baseline values.

To analyze the pH stability of the synthetic peptides, six peptide solutions (1 mg/mL) were treated for 30 min at pH 3.0, 5.0, 7.0, 9.0, and 11.0, adjusted to 7.0, lyophilized, and assayed for immunoreactivity. Samples without pH treatment served as controls and their MRPRs were compared depending on the baseline values.

### 2.12. Molecular Docking

The toll-like receptor proteins TLR2 (ID:1FYW) and TLR4/MD-2 (ID:5IJD) were retrieved from the Protein Data Bank (http://www.rcsb.org/pdb, accessed on 20 July 2022) database, and PyMOL 2.4.0 (version 2.4.0, Schrödinger, LLC, http://www.pymol.org/, accessed on 20 July 2022) was used to remove water and its own small-molecule ligands. The molecular docking of receptor proteins to ligand peptides was performed using AutoDock Vina (version 1.1.2, ADT; Scripps Research Institute, La Jolla, San Diego, CA, USA) and the binding capacity was scored.

### 2.13. Statistical Analysis

Statistical analysis was performed for each sample with triplicate data expressed as mean ± standard deviation. One-way analysis of variance (ANOVA) with SPSS software (version 26, SPSS Inc., Chicago, IL, USA) was used to compare the differences between the means of the three groups, with significant differences determined at 95% confidence intervals (*p* < 0.05).

## 3. Results and Discussion

### 3.1. Immune Activity, DH, and MW of Enzymatic Hydrolysate from L. vannamei Heads

**Figure 1:**
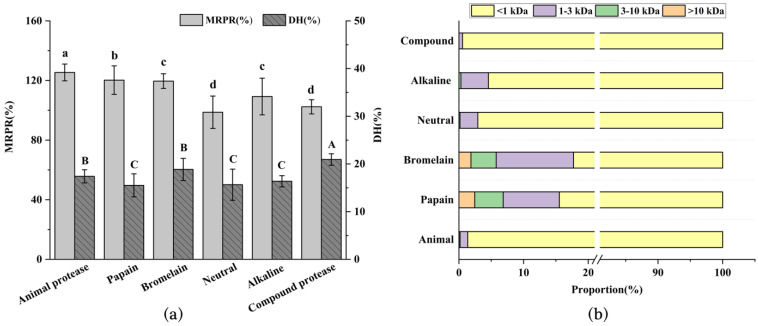
Relative proliferation rate of macrophages (MRPR) and degree of hydrolysis (DH) with different enzymatic hydrolysis products (**a**), and molecular weight distribution of different enzymatic hydrolysates (**b**). Different uppercase letters in the figure indicate significant differences in DH (*p* < 0.05); different lowercase letters indicate significant differences in MRPR (*p* < 0.05).

MRPR is the most common index to evaluate immune activity, while DH is one of the indicators to assess the effect of enzymatic hydrolysis. In this paper, six proteases were applied to the enzymolyzed *L. vannamei* heads, and the MRPR and DH of the different enzymatic hydrolysates are shown in Figure 1a. The animal protease hydrolysate exhibited the highest MRPR at 125.41 ± 5.63%, reflecting great immune activity. Meanwhile, the DH of animal protease enzymolysis product was 17.41 ± 1.38%, which was slightly lower than that of the compound protease with the highest DH. The result matched earlier studies. Xu et al. [17] used alkaline trypsin to hydrolyze *napin* and the DH of the enzymolysis product was not optimal, but this did not affect the fact that it exhibited the highest DPP-IV-inhibitory activity. These findings further supported the view that there was no close relationship between the DH and MRPR of the enzymolysis product.

In Figure 1b, the percentage of small molecules below 1 kDa in the enzymatic hydrolysates of the animal and compound proteases was much higher than that of the other enzymes. This may be attributed to the fact that both were complex enzymes, which had more cleavage sites than a single enzyme [18], making it easier to obtain short peptides with better biological activity [19]. Therefore, the selection of the appropriate protease is a crucial factor in the extraction of bioactive peptides.

### 3.2. Purification of Immunomodulatory Peptide from L. vannamei Heads

**Figure 2:**
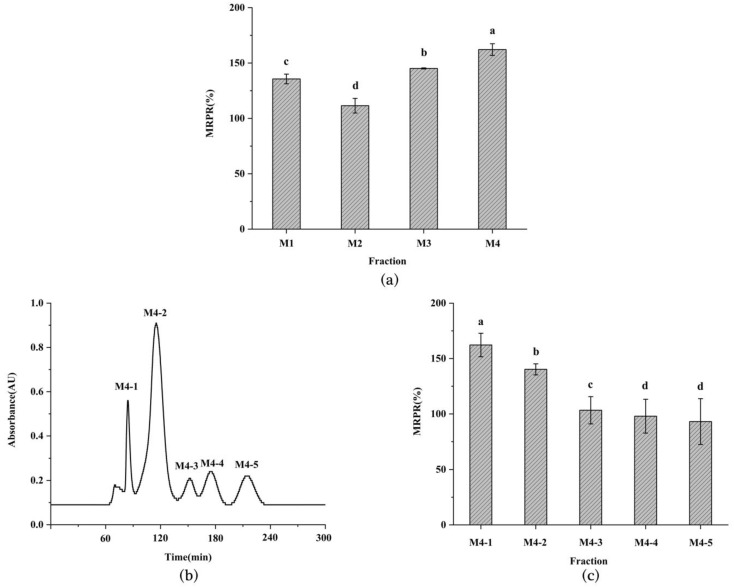
MRPR in each ultrafiltration fraction (100 μg/mL) (**a**); Sephadex G-15 gel chromatogram (**b**), and MRPR in each component (**c**). Different lowercase letters indicate significant differences in MRPR (*p* < 0.05).

Ultrafiltration is a commonly used separation technique based on the molecular weight of protein hydrolysates [20]. The *L. vannamei* heads hydrolysate was ultrafiltered to obtain four fractions. Each fraction was freeze-dried and its MRPR was measured. As can be seen from Figure 2a, the M4 fraction presented the highest MRPR (MW < 1 kDa, 162.16 ± 5.27%), significantly higher than M1 (MW > 10 kDa, 135.63 ± 4.34%), M2 (MW = 3–10 kDa, 111.47 ± 6.61%), and M3 (MW = 1–3 kDa, 145.14 ± 0.50%). This finding was in agreement with previous studies that showed that small-molecule peptides may have better bioactivity [17]. 

The fractions below 1 kDa obtained from the membrane separation of both the *Stolephorus chinensis* hydrolysate [16] and the soybean hydrolysate [21] displayed the highest immune cell activity compared to the other fractions.

To obtain the immunomodulatory peptide, the optimal fraction M4 was purified by Sephadex G-15 gel chromatography. The purification results are depicted in Figure 2b,c. Five fractions (M4-1–M4-5) of M4 were extracted by gel chromatography, with the MRPR of the M4-1 fraction being the highest (162.26 ± 10.62%) and significantly higher than the other fractions. The outcome was in agreement with the first fraction obtained by gel purification from tuna trimmings hydrolysate by Cai et al. [22] and from the enzymatic digest of Maca by He et al. [23], showing optimal immunological activity. It may be attributed the fact that the final fraction obtained by gel purification contained more free amino acids and thus reduced the immunoactivity. Therefore, the M4-1 fraction was further evaluated for immune activity.

### 3.3. Determination of Immune Activity of M4-1 Fraction

**Figure 3:**
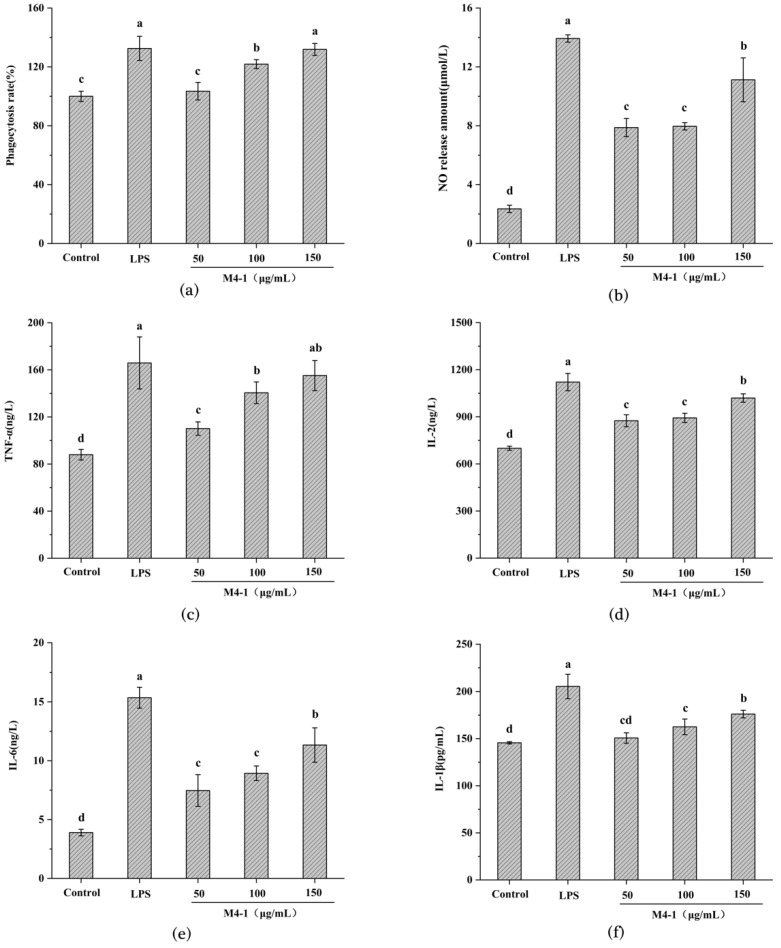
Evaluation of the immunomodulatory property of the M4-1 fraction. Effects of different concentrations of M4-1 components on phagocytosis of neutral red (**a**), NO release (**b**), and cytokines TNF-α (**c**), IL-2 (**d**), IL-6 (**e**), and IL-1β (**f**) in macrophages. Values in columns a–d with different superscripts indicate significant differences (*p* < 0.05).

Macrophage phagocytosis, which protects against infections caused by foreign biotic and abiotic attacks, is an important indicator of the immunomodulatory role of macrophages [24]. The result of the phagocytosis of neutral red was presented in Figure 3a, with a dose-dependent phagocytosis of the M4-1 fraction in the range of 50 to 150 μg/mL. The phagocytosis of macrophages was greatly strengthened at medium and high concentrations of stimulation compared to the blank group, and the phagocytosis of the M4-1 fraction at high concentrations (131.86 ± 4.07%) was equivalent to that of the LPS-promoted cells. Similarly, it had been reported that the F31 fractions purified from oyster hydrolysates improved the phagocytosis effect in a positive correlation with the concentration [25].

NO is an active mediator that plays a role in immune regulation through the activation of macrophages, and the degree of macrophage activation was assessed by measuring the amount of NO secretion. As seen in Figure 3b, there was no significant difference in NO production induced by low and medium concentrations of M4-1 fraction, but as the concentration of M4-1 fraction increased, the maximum amount of NO secretion was reached at 150 μg/mL (11.12 ± 1.49 μmol/L). This outcome indicated that the presented concentration of the M4-1 fraction upregulated the level of NO secretion in the cells, which was consistent with former research on the hydrolysis products of *Cyclina sinensis* [26] and the protein hydrolysate of *Nibea japonica* [27].

Cytokines have an essential role in the survival, proliferation, and differentiation of immune cells [28]. In Figure 3c,d, compared to the blank control, the secretion of TNF-α and IL-2 at low concentrations increased by 25.26% and elevated by 25.03%, respectively, both of which significantly enhanced their release. As presented in Figure 3e,f, IL-6 and IL-1β did not change significantly in the low and medium concentration ranges of M4-1 release, but both had significantly elevated secretion at high concentrations and reached a maximum value of 11.34 ± 1.46 ng/L for IL-6 and 176.10 ± 3.99 pg/L for IL-1β. These results revealed that the M4-1 fraction exerted immunomodulatory effects in a dose-dependent manner over a range of concentrations, which was similar to many previous studies. He et al. [29] extracted MCP from *Mytilus coruscus*, showing a dependent increase in cytokine secretion from 12.5 to 100 μg/mL. Xu et al. [16] obtained IPSCs (50–200 μg/mL) from *Stolephorus chinensis*, which increased the secretion of TNF-α, IL-6, and IL-1β. 

Previous reports have demonstrated that immunomodulatory peptides derived from food proteins are related to the MW of the peptide, the structure of the peptide, the number of characteristic amino acids, hydrophobicity, and charge [30]. Consequently, further sequence identification and screening of M4-1 for immunomodulatory peptides was required.

### 3.4. MS Identification of Immunomodulatory Peptides

The peptide sequences of the M4-1 fractions were identified by LC-MS/MS and the MS results obtained were compared with the corresponding protein database (Table S1), and then eleven peptides were screened by PeptideRanker score [31], as listed in Table 1. These peptides were analyzed with the Expasy-pI/Mw tool (https://web.expasy.org/compute_pi/, accessed on 20 June 2022) and Peptide2.0 (http://peptide2.com, accessed on 20 June 2022) to assess the isoelectric point (pI), MW, and hydrophobicity.

**Figure 4:**
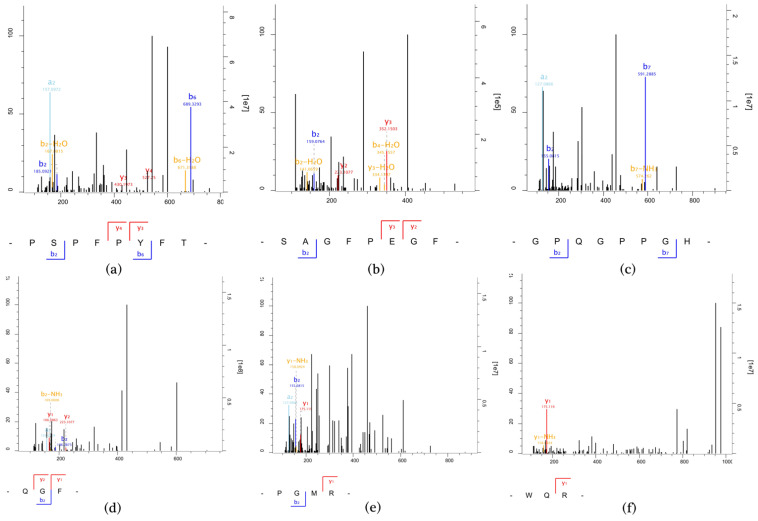
MS/MS spectra of six immunomodulatory peptides identified and screened from the M4-1 fraction PSPFPYFT (**a**), SAGFPEGF (**b**), GPQGPPGH (**c**), QGF (**d**), PGMR (**e**), and WQR (**f**).

Combined with some previously reported studies on immunomodulatory peptides [5,30], the characteristics of peptides that may contribute greatly to their immune enhancement are mainly as follows: the peptides generally consist of 2 to 20 amino acids; the majority of amino acid residues in the peptides are the hydrophobic amino acids Trp, Phe, and Ala, etc., the branched amino acids Val, Ile, and Leu, etc., and the basic amino acids Lys and Arg; and the peptides have high hydrophobicity and pI. Thus, six immunomodulatory peptides, PSPFPYFT, SAGFPEGF, GPQGPPGH, QGF, PGMR, and WQR, were selected and their immunoreactivity was further determined. The MS/MS spectra of the six peptides are shown in Figure 4.

### 3.5. Immunoactivity Assay of Synthetic Peptides

The six synthetic peptides in Table 2 possessed the ability to immunomodulate. Among them, PSPFPYFT, GPQGPPGH, PGMR, and WQR significantly increased MRPR, and showed higher phagocytosis capacity and TNF-α secretion. This also accorded with earlier articles, such as the wheat germ globulin peptide ECFSTA [32], which had good cytophagocytic capacity and enhanced secretion of NO, IL-6, TNF-α, and ROS of RAW 264.7 cells, and the *Pseudostellaria heterophylla* peptide RGPPP (100 μg/mL) [28], which had a stimulation index of 1.27 on splenic lymphocytes and upregulated TNF-α secretion to 827.24 pg/mL, and the cytokinesis rate increased to 123.81%. These peptides also increased IL-6 secretion; however, the observed difference between the various peptides was not significant, which may require further explanation through molecular mechanisms. The findings suggested that peptides from the protein of *L. vannamei* heads had the potential for immune-enhancing regulation. Moreover, the six peptide sequences obtained in the study were not present in other reported work about immunomodulatory peptides by BIOPEP (http://www.uwm.edu.pl/biochemia/index.php/en/biopep, accessed on 20 June 2022).

### 3.6. Stability Analysis of Synthetic Peptides

**Figure 5:**
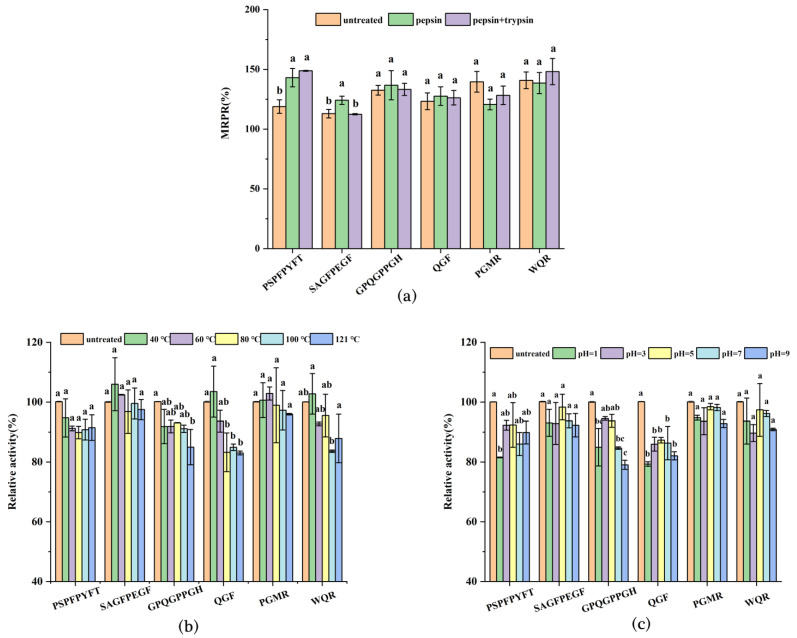
Stability analysis of synthetic peptides. Effects of gastrointestinal digestion (**a**), heat treatment (**b**), and pH treatment (**c**) on the relative proliferation rate of macrophages. Values in columns a–d with different superscripts indicate significant differences (*p* < 0.05).

The activity of bioactive peptides after being hydrolyzed by digestive enzymes in vivo is one of the most important factors affecting the application of active peptides. The changes in the peptide structures of the six immunomodulatory peptides after computer-simulated gastrointestinal digestion are shown in Table S2. The four peptides were hydrolyzed during gastric digestion. They were not cleaved during the intestinal digestion as they did not possess the trypsin cleavage site, while GPQGPPGH and PGMR failed to be cleaved during gastrointestinal digestion. Since the computer simulations theorized the enzymatic cleavage sites of pepsin and trypsin and idealized the hydrolysis process, the effect on peptide activity required further experimental verification. Figure 5a presents the results of in vitro gastrointestinal stability for the peptides. Compared with the untreated group, the MRPR of GPQGPPGH and WQR were maximally increased by 4.17% and 7.36% through gastrointestinal digestion, while the PGMR activity decreased, but was not significantly different. Similar results were obtained for antioxidant peptides in carp muscle hydrolysates [33]. Interestingly, the MRPR of PSPFPYFT was significantly raised to 143.06 ± 7.71% and 148.80 ± 0.41% after the action of pepsin and trypsin, respectively. In addition, the MRPR of SAGFPEGF increased by 11.23% in the gastric digestion. This finding was parallel to the earlier article. The DPP-IV inhibitory activity of walnut peptides was increased by gastrointestinal digestion simulations [34], and Qingke peptide continued to exert ACE inhibitory activity during gastrointestinal digestion [35]. Furthermore, according to the computer simulations, some peptides produced after gastrointestinal digestion still possessed active characteristics. For example, PSPFP and SAGF still had a high proportion of hydrophobic amino acids, and QR still held a high hydrophobicity (+10.48 Kcal mol^−1^) and isoelectric point (pI = 10.80). This may be the reason why these peptides maintained their immunoreactivity.

Active peptides are inevitably affected by the temperature in food processing, and Figure 5b demonstrated the effect of different temperature treatments of synthetic peptides on their activity. As the temperature increased from 25 °C to 121 °C, the relative proliferation rates of the six peptides had no significant effect compared with the control group. Among them, the relative activities of PSPFPYFT, SAGFPEGF, PGMR, and WQR were still higher than 90% even after treatment at 121 °C for 30 min, which may be related to the size and structure of the peptide and the proportion of hydrophobicity in the peptide. Therefore, the six peptides still exerted immune activity after being treated at different temperatures, and could withstand the thermal processing of food.

pH is one of the major factors affecting peptide activity, and the relative activity effects of different pH treatments of the six peptides are shown in Figure 5c. In the pH range of 3.0 to 11.0, the activity of the six peptides did not reduce significantly and there were only slight fluctuations. It can also be observed that in the neutral environment, the relative activity of all peptides except QGF reached over 90% of that of the untreated group. Although peptide activity was affected in the acidic and alkaline environment, the six peptides still exhibited good immune activity, achieving about 80% of that of the untreated group. This was similar to the previously reported pH stability results of ACE-inhibitory peptide DLTAGLL isolated and screened from *Lepidotrigla microptera* [36]. Therefore, six immunomodulatory peptides can be applied to food systems with pH 3.0–11.0, while performing favorable immunoreactivity.

### 3.7. Molecular Docking of Immunomodulatory Peptides

Four peptides (PSPFPYFT, GPQGPPGH, PGMR, and WQR) were selected for molecular docking analysis based on the stability and immunoreactivity. Molecular docking was exploited to study the binding patterns of peptides to the receptor proteins TLR2 (ID:1FYW) and TLR4/MD-2 (ID:5IJD) and to assess their affinity [37]. The binding scores of the peptide-TLR2 and peptide-TLR4/MD-2 complexes are listed in Table 3, indicating a weaker binding relationship between the peptide and TLR2 than TLR4/MD-2. This may be due to the mainly hydrophobic area of the TLR4/MD-2 cavity and the relatively large cavity [38]. According to the score scale, GPQGPPGH and WQR showed strong binding to TLR2, both scoring −7.3. GPQGPPGH, PSPFPYFT, and WQR exhibited high binding to TLR4/MD-2, scoring −8.7, −9.3, and −8.1, respectively. The molecular docking results were consistent with the data in Table 2. GPQGPPGH and WQR exhibited better cell proliferation rates, phagocytosis, and cytokine release compared to the other peptides. 

**Figure 6:**
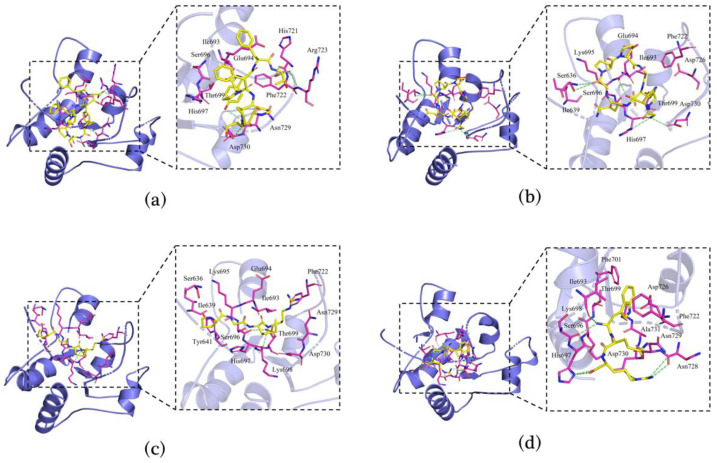
Binding and interacting residues of peptides with TLR2 (ID: 1FYW). PSPFPYFT-TLR2 (**a**), GPQGPPGH-TLR2 (**b**), PGMR-TLR2 (**c**), and WQR-TLR2 (**d**). In the figure, green represents hydrogen bonds, yellow represents polypeptide ligands, and purple represents amino acid residues related to the action.

**Figure 7:**
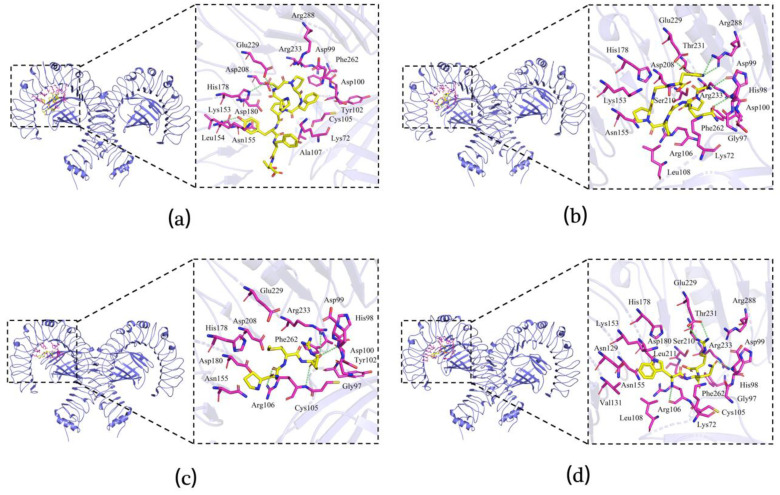
Binding and interacting residues of peptides with TLR4/MD-2 (ID: 5IJD). PSPFPYFT-TLR4/MD-2 (**a**), GPQGPPGH-TLR4/MD-2 (**b**), PGMR-TLR4/MD-2 (**c**), and WQR-TLR4/MD-2 (**d**). In the figure, green represents hydrogen bonds, yellow represents polypeptide ligands, and purple represents amino acid residues related to the action.

Hydrogen bonds were formed between the receptor and the peptides, allowing for tighter binding of the peptides. In Figure 6a–d, four hydrogen bonds were formed between PSPFPYFT and the Phe722, Arg723, Asn729, and Asp730 residues of TLR2; seven hydrogen bonds were formed between GPQGPPGH and the Ser636, Glu694, Thr699, His697, and Asp730 residues of TLR2; two hydrogen bonds were observed between PGMR and the His697 and Ser696 residues of TLR2; and seven hydrogen bonds were observed between WQR and the Ile693, Ser696, His697, Asn728, and Asp730 residues of TLR2. As shown in Figure 7a–d, three hydrogen bonds were formed between PSPFPYFT and the Leu154, His178, and Glu229 residues of TLR4/MD-2; five hydrogen bonds were formed between GPQGPPGH and the Arg288, Glu229, and Asp99 residues of TLR4/MD-2; four hydrogen bonds were observed between PGMR and the Arg106, Asp100, and Arg233 residues of TLR4/MD-2; and there were seven hydrogen bonds observed between WQR and the Thr231, Glu229, His98, and Arg106 residues of TLR4/MD-2. 

As a whole, hydrogen bond forces could be a factor in the differences in ligand–receptor interactions (which affected more than charge interactions and π-π bond interactions). Among the complexes containing more hydrogen bonds, the WQR-TLR4/MD-2 complex and the GPQGPPGH-TLR2 complex contributed to a strong binding interaction, and molecular dynamics simulations also proved the stable binding of the peptide to the protein (Supplementary Material), which allowed the peptide to show better immunoreactivity, similar to the results of the in vitro cellular assays in Table 2. These findings agreed with the results of three peptides derived from duck egg yolk [38] and HIAEEADRK and AEQAESDKK from tuna protein [22]. Hence, *L. vannamei* head peptides could regulate immunity by forming hydrogen bonds with binding sites on the TLR receptors and activating the relevant signaling pathways.

## 4. Conclusions

This study was the first to use protein from the heads of *L. vannamei* to prepare immunomodulatory peptides. The shrimp head protein was acted on by different proteases, and the highest MRPR was observed for the animal protease hydrolysate, and M4-1 fraction obtained by ultrafiltration and G15 gel chromatography exhibited the highest MRPR. The M4-1 fraction (50–150 μg/mL) could significantly promote NO production by macrophages, enhance the phagocytic ability of macrophages, remarkably increase the secretion of TNF-α, IL-2, IL-6 and IL-1β, and show a certain dose-dependent effect, exerting immunomodulatory effects. The six immunomodulatory peptides, PSPFPYFT, SAGFPEGF, GPQGPPGH, QGF, PGMR, and WQR, were identified by LC-MS/MS, and stability tests showed that these peptides maintained good immune activity under different temperatures, pH, and in vitro-simulated gastrointestinal digestion. Molecular docking elucidated the mechanism of immunomodulatory peptides through the formation of hydrogen bonds by binding to receptor proteins. The results suggested that *L. vannamei* head peptides could be used as a potential immune enhancer from natural food sources and may also provide a high-protein immune supplement for vegetarians. However, this study was limited to in vitro experimental analysis and mechanistic research, and further in vivo studies are necessary to verify whether *L. vannamei* head immunomodulatory peptides could be used as a functional supplement to enhance the immunity of the body. This paper demonstrated that *L. vannamei* head peptides possessed immune-enhancing activity, with a view to provide a theoretical basis for the high-value utilization of *L. vannamei* head protein and the development of food-derived immunomodulators.

## Figures and Tables

**Table 1:** Peptide sequences and information scoring of immunomodulatory peptides from *L. vannamei* heads.

No.	Sequence	Length	Mass	PeptideRanker	pI	Hydrophobicity
1	PSPFPYFT	8	954.44872	0.962878	5.95	62.50%
2	SAGFPEGF	8	810.35482	0.898452	4.00	50.00%
3	GPQGPPGH	8	745.35074	0.802084	6.74	37.50%
4	PGAKCYGF	8	841.37926	0.889124	8.60	37.50%
5	PGCACLPG	8	716.29857	0.903095	5.87	50.00%
6	GSGGCGHW	8	759.27586	0.862882	6.73	12.50%
7	QGF	3	350.15902	0.940972	5.52	33.33%
8	PGMR	4	459.22639	0.900324	10.18	50.00%
9	WQR	3	488.24957	0.840511	9.75	33.33%
10	YYSF	4	578.23766	0.806248	5.52	25.00%
11	WCH	3	444.15797	0.968801	6.73	33.33%

**Table 2:** Immunomodulatory activities of synthetic peptides.

Sequence	MRPR (%)	Phagocytosis Rates (%)	TNF-α (ng/L)	IL-6 (ng/L)
PSPFPYFT	126.27 ± 11.65% ^bc^	141.59 ± 2.96% ^bc^	70.70 ± 3.02 ^a^	21.63 ± 2.51 ^a^
SAGFPEGF	111.26 ± 0.77% ^cd^	138.99 ± 6.64% ^bc^	54.69 ± 1.46 ^b^	25.45 ± 1.63 ^a^
GPQGPPGH	150.17 ± 1.92% ^a^	159.41 ± 5.51% ^a^	56.24 ± 5.83 ^b^	21.94 ± 1.03 ^a^
QGF	107.67 ± 9.60% ^cd^	129.27 ± 9.58% ^c^	62.41 ± 3.01 ^ab^	25.98 ± 1.90 ^a^
PGMR	143.52 ± 0.76% ^ab^	148.12 ± 2.03% ^ab^	60.06 ± 7.23 ^b^	21.12 ± 3.69 ^a^
WQR	134.69 ± 15.23% ^ab^	156.59 ± 9.15% ^a^	56.18 ± 1.41 ^a^	22.22 ± 1.09 ^a^
Control	100.00 ± 0.02% ^d^	100.18 ± 0.07% ^d^	39.10 ± 2.33 ^c^	13.80 ± 2.00 ^b^

* Synthetic peptide acting cells were measured for their MRPR and phagocytosis rates (100 μg/mL) and for IL-6 and TNF-α release (150 μg/mL). Different letters in a column represent significant differences between groups for multiple range analysis (*p* < 0.05).

**Table 3:** Molecular docking analysis of the peptides by AutoDock Vina.

Sequence	Affinity (kcal/mol)	
	TLR2 (ID: 1FYW)	TLR4/MD-2 (ID: 5IJD)
PSPFPYFT	−7.0	−9.3
GPQGPPGH	−7.3	−8.7
PGMR	−6.8	−6.8
WQR	−7.3	−8.1

## Data Availability

The authors confirm that the data supporting the findings of this study are available within the article or supplementary material.

## Supplementary Materials

The following supporting information can be downloaded at: https://www.mdpi.com/article/10.3390/foods11203309/s1, Figure S1: Graphical representation of the molecular dynamics simulation studies conducted during 100000 ps. Root mean square deviation (RMSD) (a). Root mean square fluctuation (RMSF) of GPQGPPGH-TLR2(b). RMSF of WQR-TLR4/MD-2(c). Radius of gyration (Rg) curves(d). Hbond number of the protein(e); Table S1: The parent protein information of the eleven immunomodulatory peptides; Table S2: Immunomodulatory peptides from the heads of Litopenaeus vannamei gastrointestinal digestion ("/" indicates protease cleavage site).; Table S3: Binding free energies and energy components predicted by MM/GBSA (kcal/mol).
