# Supplementary material for: Purification, Identification and Molecular Docking of Immunomodulatory Peptides from the Heads of Litopenaeus vannamei
Source: Foods. 2022 Oct 21;11(20):3309. doi: 10.3390/foods11203309 (PMC9602407; doi:10.3390/foods11203309)
Supplement: Supplementary file 1 [file foods-11-03309-s001.zip › foods-1930134-supplementary.pdf]

Supplementary materials:

Table S1. The parent protein information of the eleven immunomodulatory peptides.

Sequence	leading razor protein	Protein name	Gene name	Score(MS/MS)
PSPFPYFT	A0A3R7NRD4	Uncharacterized protein	C7M84_017414	43
SAGFPEGF	A0A3R7MRM8	Uncharacterized protein	C7M84_013796	27.121
GPQGPPGH	A0A3R7N7F6	Putative collagen alpha-2(IX) chain-like isoform X2	C7M84_002020	22.622
PGAKCYGF	A0A423TNK8	Putative scaffold attachment factor B2-like	C7M84_003253	17.767
PGCACLPG	A0A3R7LQ73	Uncharacterized protein	C7M84_020737	10.185
GSGGCGHW	A0A3R7QNB8	Suppressor of cytokine signaling 5	C7M84_022259	1.619
QGF	A0A3R7PSP3	Dynein heavy chain, cytoplasmic	C7M84_025389	21.978
PGMR	A0A423TWP3	Putative histone-lysine N-methyltransferase 2C-like isoform X2	C7M84_000393	18.534
WQR	A0A3R7Q852	Putative dynein heavy chain 5, axonemal-like isoform X2	C7M84_010724	18.31
YYSF	A0A423TWS3	KASH domain-containing protein	C7M84_000350	6.6306
WCH	A0A3R7PSP3	Dynein heavy chain, cytoplasmic	C7M84_025389	9.6433E-16

Tableb S2. Immunomodulatory peptides from the heads of *Litopenaeus vannamei* gastrointestinal digestion ("/" indicates protease cleavage site).

Sequence	Pepsin（pH>2）		Trpsin	
	Positions of cleavage sites	Stomach digestion	Positions of cleavage sites	Intestinal digestion
PSPFPYFT	5 7	PSPFP/YF/T	5 7	PSPFP/YF/T
SAGFPEGF	4 7 8	SAGF/PEG/F	4 7 8	SAGF/PEG/F
GPQGPPGH	/	/	/	/
QGF	2 3	QG/F	2 3	QG/F
PGMR	/	/	/	/
WQR	1	W/QR	1	W/QR

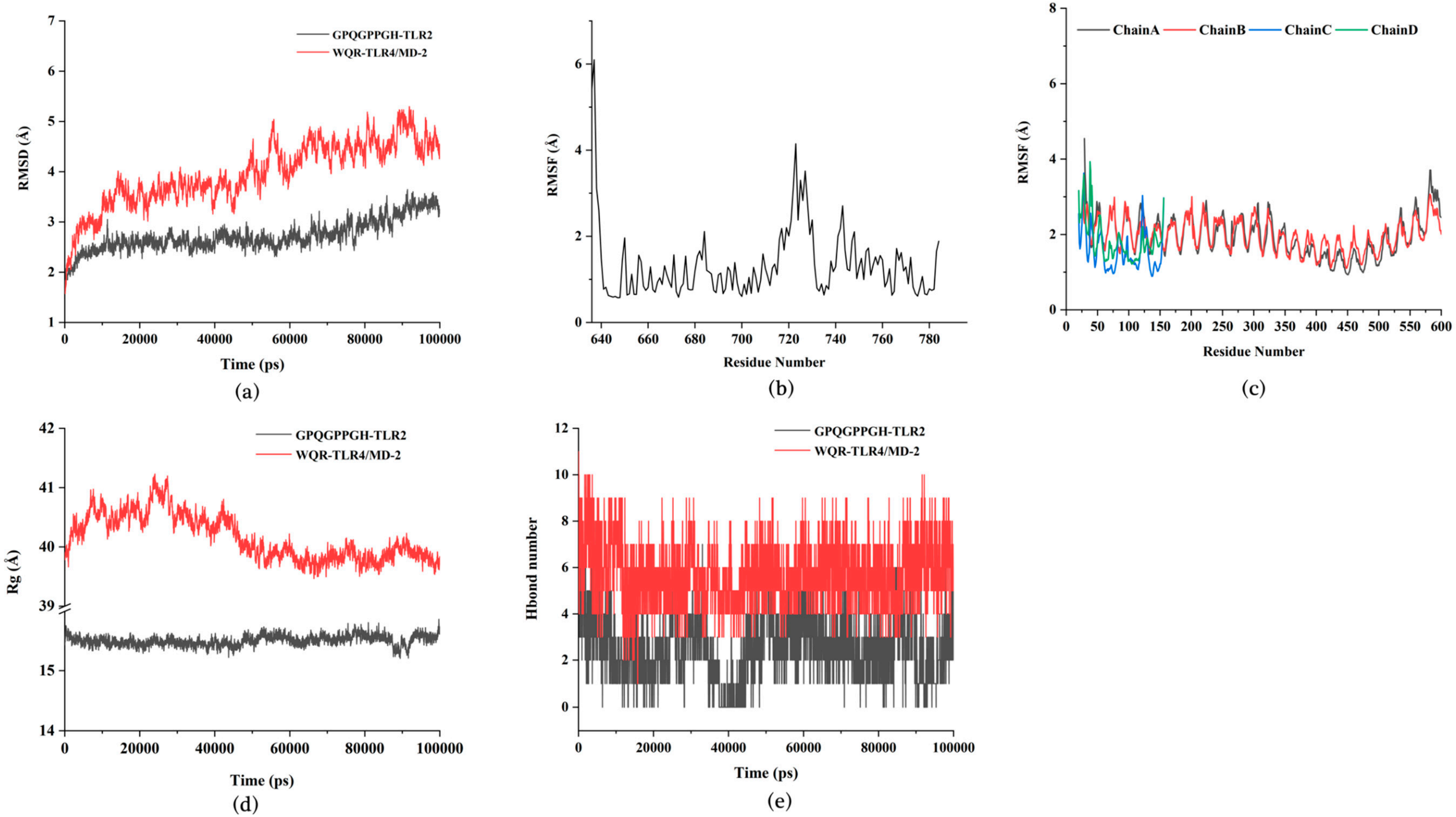

Figure S1. Graphical representation of the molecular dynamics simulation studies conducted during 100000 ps. Root mean square deviation (RMSD) (a). Root mean square fluctuation (RMSF) of GPQGPPGH-TLR2(b). RMSF of WQR-TLR4/MD-2(c). Radius of gyration (Rg) curves(d). Hbond number of the protein(e).

As can be seen from Figure S1a, the binding of two peptides to the protein had some effects on the protein structure, with the protein conformation fluctuating to some extent, but both were stable after 60,000 ps. The results indicated that the binding of the small molecule to the receptor protein resulted in a slight change in its conformation, but the overall conformation was stable. The small molecule formed a tightly bound complex with the protein.

The RMSF curves represented the fluctuations of the amino acid residues of the protein. In Figure S1b, the RMSF of GPQGPPGH-TLR2 was mostly below 2 Å, showing that this protein system remained relatively stable, especially in the segment with the sequence number of 640-710. Figure S1c shows the RMSF for the four chains in the WQR-TLR4/MD-2 system. Most of the RMSF were around 2 Å, with a minimum value of 0.9341 Å and a maximum value of 6.1328 Å, which indicated that several smaller regions of the core structural domain of the protein provided greater flexibility compared to other regions.

The Rg curves represented the compactness of the whole protein structure. It can be seen from Figure S1d that the Rg of GPQGPPGH-TLR2 and WQR-TLR4/MD-2 were stab le around 15.8 Å and 40 Å, and the latter fluctuated relatively more, which might be rela ted to more binding sites of WQR and TLR4/MD-2. However, in general, the binding of two small molecules to corresponding receptors had no significant effect on protein foldin g.

In molecular docking studies, hydrogen bonds had been shown to be the main binding force between immunomodulatory peptides and TLR2 and TLR4/MD-2 proteins. Figure 1e shows the number of hydrogen bonds formed between amino acid residues of peptides and receptor proteins with simulation time. GPQGPPGH-TLR2 formed 0-8 hydrogen bonds and most of the time 2-5 hydrogen bonds, while WQR-TLR4/MD-2 formed 1-11 hydrogen bonds and most of the time 4-8 hydrogen bonds, demonstrating that both of them were mainly bound by hydrogen bonding interactions.

Table S3. Binding free energies and energy components predicted by MM/GBSA (kcal/mol).

Energy Component	GPQGPPGH-TLR2	WQR-TLR4/MD-2
ΔGvdw	-36.4122	-40.2417
ΔGele	-57.3621	-89.2196
ΔGpolar	77.6199	102.2512
ΔGnonpolar	-4.9766	-6.1348
ΔGgas	-93.7743	-129.4613
ΔGsolv	72.6433	96.1164
ΔGtotal	-21.1311	-33.3449

Notes: ΔGvdw, van der waals energy. ΔGele, electrostatic energy. ΔGpolar, polar contribution to solvation. ΔGnonpolar, non-polar contribution to solvation. ΔGgas, molecular mechanics term (energy in gas phase). ΔGsolv, solvation energy. ΔGtotal, binding free energy.

Table S3 presents the calculated binding free energy between small molecules and proteins based on the MMGBSA equation (50-100 ns trajectories were taken for calculation). The results showed that the binding free energy of GPQGPPGH-TLR2 was -21.1311 kcal/mol. ΔGvdw (-36.4122 kcal/mol) and ΔGele (-57.3621 kcal/mol) favored the binding, while ΔGpolar (77.6199 kcal/mol) disfavored the interaction of the two. The binding free energy of WQR-TLR4/MD-2 was -33.3449 kcal/mol. ΔGvdw (-40.2417 kcal/mol) and ΔGele (-89.2196 kcal/mol) favoured the binding, while ΔGpolar (102.2512 kcal/mol) was unfavourable. The WQR-TLR4/MD-2 had a lower binding energy compared to GPQGPPGH-TLR2, which also suggested a better binding ability between WQR and the receptor protein.

Based on the findings of molecular docking, the systems GPQGPPGH-TLR2 and WQR-TLR4/MD-2 were selected for molecular dynamics simulations. The results were analysed by parameters of RMSD, RMSF, Rg and hydrogen bond formation, and it was concluded that the peptide ligands were able to form stable complexes with the target protein receptors. In addition, MM/GBSA analysis obtained the binding free energy for both complexes, which could also indicate stable binding.
